# The complete mitochondrial genome of Purple Spot Mantis Shrimp *Gonodactylus smithii* (Pocock, 1893)

**DOI:** 10.1080/23802359.2021.1942272

**Published:** 2021-06-21

**Authors:** Mingqiu Yang, Hongtao Liu, Rong Wang, Wei Tan

**Affiliations:** a Key Laboratory of Utilization and Conservation for Tropical Marine Bioresources (Hainan Tropical Ocean University), Ministry of Education, Sanya, China; b Hainan Provincial Key Laboratory of Tropical Maricultural Technologies, Hainan Academy of Ocean and Fisheries Sciences, Haikou, China

**Keywords:** *Gonodactylus smithii*, mitochondrial genome, phylogenetic analysis

## Abstract

In this study, the whole mitochondrial genome of the Purple Spot Mantis Shrimp *Gonodactylus smithii* from the South China Sea was determined using next-generation sequencing. The circular mitogenome of *G. smithii* is 16,260 bp long and consists of 13 protein-coding genes (PCGs), 22 tRNA genes, and two rRNA genes. The base composition is AT-rich and has an overall AT content of 67.76% (composition of A, G, T, and C was 35.30%, 12.41%, 32.46%, and 19.83%, respectively). Among 13 PCGs, 12 PCGs shared a common ATN as the start codon except *COX1* gene using an abnormal putative first codon GCG. 11 PCGs ended with TAA or TAG, while *ND6*, *COX2* gene terminated with a single T and *ND3* gene used a special “GAT” as the stop codon. The phylogenetic tree showed that *G. smithii* was clustered with *Gonodactylus chiragra*, then together with *Gonodactylaceus randalli*.

As a member of the family Gonodactylidae, *Gonodactylus smithii* (Pocock 1893) commonly known as purple spot mantis shrimp or Smith's mantis shrimp is a species of mantis shrimp of the smasher type which even can be capable of cracking/breaking aquarium glass. The purple spot mantis shrimp is native to a range in the Indo-Pacific Ocean extending from the Australian region through India to eastern Africa (Caldwell and Dingle 1975). It has been occupied coral reef flats ranging from low intertidal depths as low as less than 1 m–60 m, but are most common in the low intertidal depths (Caldwell and Dingle 1975). One benefit that purple spot mantis shrimp provide to humans is colorful and fascinating additions to aquariums. Till now mitochondrial genome records of Stomatopod species are inadequate. In the family Gonodactylidae of Stomatopod, only *Gonodactylus chiragra* was reported (Swinstrom et al. 2005). As of now, the molecular studies found low support for their estimates of the deep relationships among stomatopods (Ahyong and Jarman 2009; Porter et al. 2010). In the present study, we provide the complete mitochondrial genome analysis of the purple spot mantis shrimp which would provide additional data for understanding the evolution of mitochondrial genomes of the class.

Samples of the purple spot mantis shrimp were collected from Xiaodonghai, Sanya, China (N18°12′51.3396″, E109°30′31.806″), and stored at the marine crustacean specimen room (Zhide Fu, 903385534@qq.com) under the voucher number C20190927GS in Qionghai research base of Hainan Academy of Ocean and Fisheries Sciences. The library with an average length of 350 bp was constructed using the NexteraXT DNA Library Preparation Kit (Illumina Ltd, USA), and sequencing was performed on the Illumina Novaseq platform (Total Genomics Solution Limited, SZHT) the 150 bp average length of the generated reads. The whole mitochondrial genome was assembled with 2.61 G clean reads using the de novo assembler SPAdes 3.11.0 (Bankevich et al. 2012) and annotated using the MITOS (http://mitos.bioinf.uni-leipzig.de/index.py). The phylogenetic analysis was carried out based on the 13 PCGs encoded by 16 Malacostraca mitogenomes available in GenBank using IQ-TREE v1.6.12 (Nguyen et al. 2015) by maximum likelihood (ML) method with 1000 bootstrap replicates, the mtMet + F+R5 was chosen as the best-fit model according to Bayesian information criterion (BIC).

The complete mitogenome of the purple spot mantis shrimp (Accession no: MW574903) is a circular molecule of 16,260 bp in length. The base composition is 35.3% A, 12.41% G, 32.46% T, and 19.83% C. Like that reported for other arthropod genomes, it is AT-rich and has an overall AT content of 67.76%. It contains 13 protein-coding genes (PCGs), 22 tRNA, two rRNA. Four PCGs (ND1, ND4, ND4L, and ND5), eight tRNA, and two rRNA genes were located on the light strand, the others were encoded by the heavy strand.

The 22 tRNA genes in the mitogenome of the purple spot mantis shrimp vary from 64 bp to 71 bp. Two tRNA were present more than once: tRNA-Leu and tRNA-Ser both have two type copies respectively. The 12S rRNA was 841 bp long and located between tRNA-Val and tRNA-Ile. the 16S rRNA is 1,366 bp long and located between 12S rRNA and tRNA-Leu. There were 11 overlapping regions of 1-7 bp in length. The two longest overlapping regions were respectively located between *ND4* and *ND4L*, *ATP8* and *ATP6*. There were 13 intergenic sequences in the mitochondrial genome varying from 1 to 1347 bp in length. The largest intergenic sequence was located between 12S rRNA and tRNA-Ile (Supply Table S1). Among 13 PCGs, 12 PCGs shared a normal ATN as the start codon except *COX1*. The putative first codon of the *COX1* gene is GCG. The lack of a standard initiation codon in *COX1* genes is common in arthropod mitochondria (Cook 2005). 11 PCGs ended with TAA or TAG, while *ND6*, *COX2* gene terminated with a single T; *ND3* used a special “GAT” as the stop codon.

The phylogenetic tree (Figure 1) showed that *G. smithii* was clustered with *G. chiragra*, then together with *Gonodactylaceus randalli*. Our results further revealed the taxonomic and evolutionary position of Gonodactylidae in Stomatopoda. The results are largely consistent with previous studies based on three mitochondrial and two nuclear markers (Van Der Wal et al. 2017). Taken together, the newly sequenced mitochondrial genome of *G. smithii* characterized here should contribute to a better understanding of phylogenetic relationships of stomatopods species, and molecular identification, population genetic and evolutionary biological studies of the purple spot mantis shrimp.

**Figure 1.**
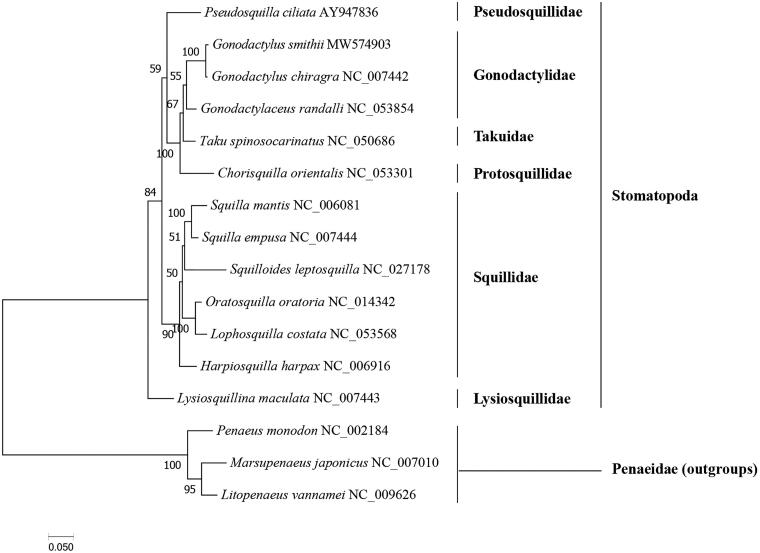
Phylogenetic tree *G. smithii* and 15 other species based on the 13 PCGs using ML methods.

The GenBank accession number for each species is indicated after the scientific name. The ML tree was carried out using IQ-TREE v1.6.12 with 1000 bootstrap replicates, the best-fit model is mtMet + F+R5. The bootstrap values were labeled at each branch node. *Litopenaeus vannamei*, *Penaeus monodon*, and *Marsupenaeus japonicus* were used as outgroups.

## Data Availability

The genome sequence data that support the findings of this study are openly available in GenBank of NCBI at (https://www.ncbi.nlm.nih.gov/) under the accession no. MW574903. The associated BioProject, SRA, and Bio-Sample numbers are PRJNA720108, SRR14153122, and SAMN18633487 respectively.
